# Occurrence of Volcanogenic Inorganic Mercury in Wild Mice Spinal Cord: Potential Health Implications

**DOI:** 10.1007/s12011-021-02890-0

**Published:** 2021-08-20

**Authors:** A. Navarro-Sempere, M. García, A. S. Rodrigues, P. V. Garcia, R. Camarinho, Y. Segovia

**Affiliations:** 1 Department of Biotechnology, Faculty of Science, University of Alicante, Apartado 99, 03080 Alicante, Spain; 2 Faculty of Sciences and Technology, University of the Azores, 9501-801 Ponta Delgada, Portugal; 3 IVAR, Research Institute for Volcanology and Risk Assessment, University of the Azores, 9501-801 Ponta Delgada, Portugal; 4 cE3c, Centre for Ecology, Evolution and Environmental Changes, and Azorean Biodiversity Group, University of the Azores, 9501-801 Ponta Delgada, Portugal

**Keywords:** Autometallography, Environmental pollutants, Heavy metals, Neurotoxicity, *Mus musculus*, Motor neurons, Axons, Motor neuron diseases

## Abstract

Mercury accumulation has been proposed as a toxic factor that causes neurodegenerative diseases. However, the hazardous health effects of gaseous elemental mercury exposure on the spinal cord in volcanic areas have not been reported previously in the literature. To evaluate the presence of volcanogenic inorganic mercury in the spinal cord, a study was carried out in São Miguel island (Azores, Portugal) by comparing the spinal cord of mice exposed chronically to an active volcanic environment (Furnas village) with individuals not exposed (Rabo de Peixe village), through the autometallographic silver enhancement histochemical method. Moreover, a morphometric and quantification analysis of the axons was carried out. Results exhibited mercury deposits at the lumbar level of the spinal cord in the specimens captured at the site with volcanic activity (Furnas village). A decrease in axon calibre and axonal atrophy was also observed in these specimens. Given that these are relevant hallmarks in the neurodegenerative pathologies, our results highlight the importance of the surveillance of the health of populations chronically exposed to active volcanic environments.

## Introduction

Heavy metals occur naturally in the environment from geogenic or anthropogenic sources. Weathering of metal-bearing rocks and volcanic eruptions is natural sources of heavy metals. On the other hand, the anthropogenic share of heavy metals in the environment has increased due to the global level of industrialization and urbanization [1].

Volcanoes and geothermal areas are associated with emissions of a variety of gases, such as sulphur dioxide (SO_2_), sulphuric acid (H_2_SO_4_), hydrogen sulphide (H_2_S), hydrogen chloride (HCl), hydrogen fluoride (HF), carbon dioxide (CO_2_) and the radioactive gas radon (Rn), in addition to heavy metals, such as arsenic (As), mercury (Hg), aluminium (Al), rubidium (Rb), lead (Pb), magnesium (Mg), copper (Cu) and zinc (Zn) [2, 3]. Even in the absence of conspicuous volcanic activity, many volcanoes emit significant amounts of potentially toxic elements [4], derived mainly from geochemical processes that occur during inactive periods and even in volcanoes considered extinct [5–7]. In this regard, little is known about the health effects of exposure to volcanic environments, particularly during the quiescent phases of the volcano. Several hydrothermal manifestations, such as fumarolic fields and soil degassing occur during the quiescent phases. Many populated hydrothermal regions have been identified worldwide, such as the Azores Islands [8–10], Miyakejima Island [11, 12], Hawaii [13], Iceland [14] and New Zealand [15].

### Mercury and Health

In general, heavy metals include essential metals, that are relevant for normal biological functioning, and non-essential metals (e.g. Hg). An inadequate concentration of any of them can alter normal biological functions and cause different cellular stress responses, producing reactive oxygen species and altering the structure and function of proteins [16]. This cellular toxicity can cause harmful effects on the nervous, digestive, respiratory, immune, reproductive and urinary systems. Among the heavy metals, mercury is highly toxic due to its high affinity for sulphhydryl groups present in proteins with enzymatic activity, transport and structural functions expressed in different tissues. Therefore, it is one of the heavy metals that cause many diseases. In fact, according to the WHO [17], Hg is one of the top 10 chemical agents of public health concern and “recent studies have suggested that mercury may not have a threshold below which some adverse effects do not occur”.

Mercury is considered one of the main hazardous environmental pollutants since it circulates widely in ecosystems and is not eliminated by the environment. Mercury toxicity and its neurodegenerative effects have been confirmed in several studies [18]. Chemically, mercury exists in several forms, all of which are toxic: elemental mercury (or metallic mercury, Hg^0^), inorganic mercury compounds and organic mercury compounds. The different forms of mercury often determine the route of exposure, absorption, distribution and toxicity to target organs. It is released into the environment by volcanic eruptions or human activities. Volcanic eruptions can increase the atmospheric source of mercury by 4–6 times [19]. Natural sources, such as volcanoes, are responsible for about half of the atmospheric mercury emissions [20].

The Azores archipelago (Portugal) has nine volcanic islands, some of which with active volcanism. Volcanic activity is marked by soil degassing, carbon dioxide springs and fumarolic fields. The largest of the islands, São Miguel, has three major active volcanoes: Sete Cidades, Fogo and Furnas. The Furnas volcano is estimated to release approximately 968 tons of CO_2_ per day through soil degassing [21].

In addition to gases, the volcanic environment of Furnas releases several toxic metals that are bioavailable for both animals and humans. Bagnato et al. [22] estimated that the output of gaseous elemental mercury (Hg^0^) in Furnas hydrothermal areas is 9.6 × 10^−5^ t d^−1^, for a study area of 0.04 km^2^; nevertheless, according to these authors, these values are below WHO’s threshold. Amaral et al. [23] found that Furnas’ resident mice showed a significantly higher load of Al, Pb, Zn and cadmium (Cd) in lung, liver or kidney than the animals captured in Rabo de Peixe or other heavily contaminated areas. Likewise, Ferreira et al. [24] reported that Furnas mice have higher loads of several metals, including Cd, Pb, Hg, nickel (Ni) and chromium (Cr) when compared to individuals from Rabo de Peixe. According to these authors, the mercury load in the tails of Furnas male mice was 1.8 × higher when compared to Rabo de Peixe individuals (42 vs 23 μg kg^−1^ dry wt, respectively).

It is important to note that in the case of a spatially localized threat, such as volcanoes, the physical proximity of a population to the source of the hazard is a key component of vulnerability. According to research published by Freire et al. [25], more than 8% of the world’s population in 2015 lived within 100 km of a volcano with at least one significant eruption, and more than 1 billion people (14.3%) lived within 100 km of a Holocene volcano. In addition, the human population in these areas has increased since 1975 above the rate of global population growth. Despite the large amount of the world’s population living near a volcano, few studies have been carried out on the effects of volcanogenic contamination on the central nervous system (CNS). In this regard, our previous study on mice [26] showed that chronic exposure to a volcanic environment causes accumulation of mercury in the brain, warning of possible risks to human health. The authors alert to the likely risk of increased neurodegenerative diseases in humans inhabiting these environments. Despite these findings, there are still no studies of exposure to active volcanic environments and their effects on other parts of the CNS, such as the spinal cord.

Considering all the above, in this study, we compared the presence of volcanogenic inorganic mercury in the spinal cord (in white and grey matter and vascular tissue) of mice chronically exposed to an active volcanic environment (Furnas village) with individuals not exposed (Rabo de Peixe village). The presence of volcanogenic inorganic mercury in the spinal cord of mice was determined by the autometallographic silver enhancement histochemical method. A quantification and a morphometric analysis of the ventral horn axons (axon size and roundness) were also carried out in both groups.

## Materials and Methods

### Specimen Collection

This study was carried, as in previous studies [26, 27], using a bioindicator species, *Mus musculus* since it is found in active and inactive volcanic environments and shares the habitat with humans. Two separate groups of mice were caught alive in two different areas on São Miguel Island: a volcanically active site, Furnas village, and a reference site, Rabo de Peixe village. Rabo de Peixe (with 8000 inhabitants) is a village that does not present any types of volcanic manifestations since the seventieth century [28] and, also, where there are no apparent sources for air pollution. Furnas village, with a rural population of about 1500 inhabitants, is located inside a volcanic crater with actively degassing grounds, fumarolic fields and hydrothermal vents. In this regard, several investigations have pointed out that active outgassing zones contribute to the continuous input of volatile metals into the atmosphere [3, 22, 29].

Fourteen mice (Furnas, *N* = 7; Rabo de Peixe, *N* = 7) were captured alive using traps located in human-inhabited houses at both locations as it was carried out by Navarro-Sempere et al. [26] and Navarro et al. [27]. The mice were kept alive for the time strictly necessary before euthanasia with isoflurane. Eight individuals intended for mercury detection by autometallography were then perfused through the left ventricle with a 4% paraformaldehyde fixative solution in 0.1 M phosphate buffer saline (PBS), while those who were to be used for axon counting (six individuals) were perfused with a fixative solution of 2% paraformaldehyde and 2.5% glutaraldehyde in 0.1 M PBS. After perfusion, the spinal cord was removed, and segments from cervical, thoracic and lumbar regions were postfixed overnight in the same fixative solutions before processing for microscopy. For this investigation, only the spinal cord of the lumbar segments was used.

In addition, weight and age were obtained for each individual. The age of the mice was determined according to dry crystalline lens mass, as described by Quere and Vicent [30]

Experimental procedures were approved by the Ethics Committee of the University of Azores (REF: 10/2020). All procedures were performed conformed with the recommendations of the European Convention for the Protection of Vertebrate Animals used for Experimental and Other Scientific Purposes (ETS 123), directive 2010/63EU and Portuguese law decree (DL 113/2013).

### Spinal Cord Autometallography

The spinal cord of eight individuals (Furnas, *N* = 4; Rabo de Peixe, *N* = 4) was embedded in paraffin, and tissue blocks were sectioned on the microtome Microm HM 340 E (Microm, Walldorf, Germany) at 4 μm. Serial sections were mounted on gelatinized slides.

The autometallographic (AMG) procedure was described by Danscher and Moller-Madsen [31]. Briefly, paraffin sections were dewaxed in xylene, hydrated in ethanol–water mixtures. Once hydrated, they were pretreated for 2 h in a 1% potassium cyanide solution to avoid unspecific binding during AMG. Later, sections were placed in a physical developer consisting of 50% gum Arabic, citrate buffer, hydroquinone and silver nitrate for 80 min at 26 °C in the dark. After several times of washing, the excess silver was removed by immersing the sections in Farmer’s solution at 1% for 2 min. Sections were counterstained with haematoxylin and viewed under a Leica DMRB light microscope (Wetzlar, Germany). Photomicrographs were taken with a Lumenera Infinity microscope camera (Microsercon, SLU, Madrid, Spain).

### Morphometric Analysis and Quantification of Axons

The spinal cord of six mice (Furnas, *N* = 3; Rabo de Peixe, *N* = 3) was postfixed in 1% osmium tetroxide for 1 h, washed three times in 0.1 M phosphate buffer, dehydrated in a graded series of alcohol and embedded in Epon 812 (Electron Microscopy Sciences, Hatfield, PA, USA). Transverse sections, 0.5 μm thick, were cut using a glass knife on a Leica LKB-III ultramicrotome (Leica EZ4D, Wetzlar, Germany) and stained with 1% toluidine blue.

**Fig. 1:**
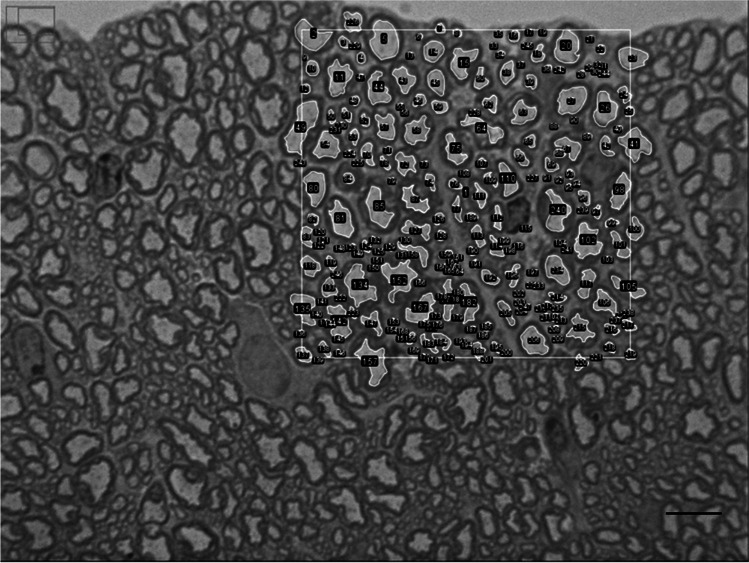
Representative image of the region of interest (ROI) in a semithin section of the ventral horn white matter. The squared zone with an area of 2000 μm^2^ represents the ROI, was selected randomly, and all the axons inside were numbered and measured; scale bar: 10 μm

For morphometric analysis, three images of ventral horn axons from each animal (*N* = 3 for each site) were captured using a Lumenera Infinity microscope camera (Microsercon, SLU, Madrid, Spain) mounted on a Leica DMRB light microscope using 100 × objective. A region of interest (ROI), with 2000 μm^2^ (Fig. 1), was randomly selected in each picture, and all axons which axoplasm fell inside each ROI were numbered. Each axoplasm was measured manually using the ROI manager tool from ImageJ software [32] to quantify the following parameters: axonal perimeter and area and aspect ratio between each other to determine the axonal roundness. The diameter value was calculated by dividing the perimeter of the axoplasm by *π*. A measure of the axoplasm roundness (*R*) or non-circularity of each axon was derived from the axoplasm perimeter and area, as described by Stankovic [33].

### Statistical Analysis

Statistical analysis was performed by Student’s *t*-test when the data were normally distributed and had equal variances. Otherwise, the Wilcoxon Mann–Whitney test was used. The level of significance was set at *p* ≤ 0.05. Graph Pad Prism was used to perform all statistical analyses (Graph Pad Software Inc., La Jolla, CA, USA), except for the weight and age of the animals, for which we have used the SPSS Statistics V. 25.0 software (IBM Corp. Armonk, NY, USA).

## Results

The samples used for the AMG did not show statistical differences in either age or weight between the studied groups. The average (± SE) age (in days) of mice was 229 ± 19 in Furnas village and 238 ± 13 in Rabo de Peixe: *t* (6) =  − 0.411, *P* = 0.695 (Student’s *t*-test). The mean (± SE) weight (in grams) was 14.96 ± 1.44 for Furnas village mice and 15.78 ± 0.95 for Rabo de Peixe mice: *t* (6) =  − 0.475, P = 0.652 (Student’s *t*-test).

Similarly, when comparing the individuals used for the morphometric analyses, there were no significant differences between the 2 groups in terms of age or weight. The mean (± SE) age (in days) was 211 ± 20 in Furnas village and 201 ± 1 in Rabo de Peixe: *t* (4) = 0.469, *P* = 0.663 (Student’s *t*-test). The mean (± SE) weight (in grams) was 13.65 ± 1.5 in Furnas village and 12.74 ± 1.24 for Rabo de Peixe mice: *t* (4) = 0.465, *P* = 0.666 (Student’s *t*-test).

### Spinal Cord Autometallography

**Fig. 2:**
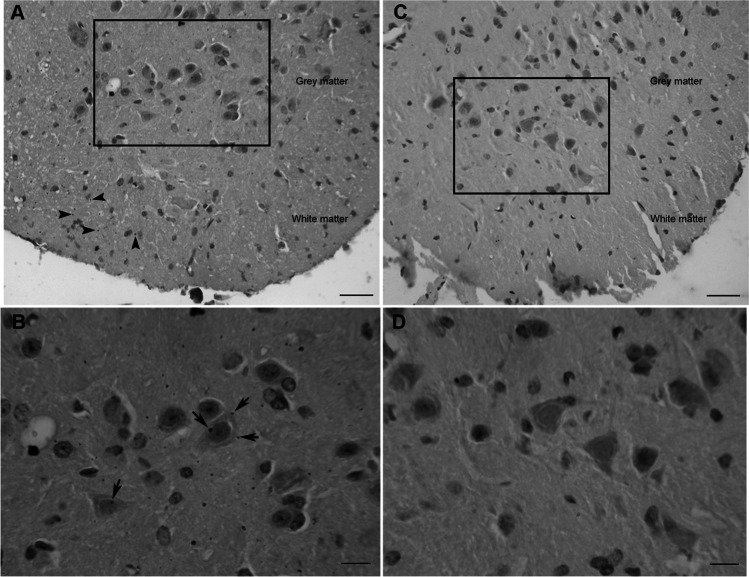
Mercury accumulation at a lumbar level in the spinal cord of chronically exposed mice (**A**–**B**) and animals from the control site (**C**–**D**). **A** AMG staining in white matter (arrowheads) and grey matter; scale bar: 25 μm. **B** Magnified microphotography from the squared area in **A**. Mercury deposit could be observed adhered to the membrane of different motor neurons (arrows); scale bar: 10 μm. **C** Lumbar segment of the spinal cord of a mouse from Rabo de Peixe. Note that there is no AMG staining in both grey and white matter; scale bar: 25 μm. **D** Inset from the squared zone in **C** where no mercury deposits are present in the grey matter of rodents from de control site; scale bar: 10 μm

Black grains were found in both grey and white matter of the spinal cord of those individuals chronically exposed to volcanic environment. By contrast, the mice living in Rabo de Peixe did not show AMG staining compatible with mercury deposits. In the white matter of Furnas mice, a disperse scattered pattern of the black grains can be observed (Fig. 2A). For this same group, in the grey matter, mercury deposits adhered to both plasma and nuclear membrane in certain cells of the ventral horn. These cells were compatible with motor neurons (Fig. 2B) according to the criteria of their localization, size and shape: large in size, multipolar and polygonal cell body with a single and prominent nucleolus. These grains are compatible with mercury deposits in these spinal cord cells. Rabo de Peixe did not show such black grains (Fig. 2C, D). To be noted, no granular mercury deposits were found in other neuronal types of glial cells in the group exposed to volcanic activity nor in the reference one.

**Fig. 3:**
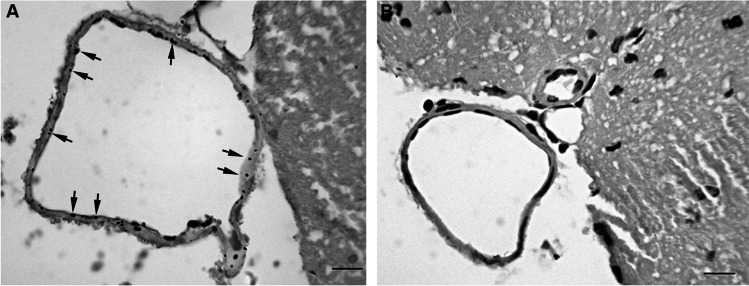
**A** Scattered pattern of mercury deposits in ventral fissure vein (arrows) in Furnas mice. **B** Ventral fissure vein from a Rabo de Peixe animal without AMG staining; scale bar: 10 μm

As in the white matter, a scattered stain pattern was found in the endothelium of the ventral fissure vein in animals exposed to the volcanic environment, but no deposits were present in the rodents from the reference site (Fig. 3).

### Morphometric Analysis and Quantification of Axons

In the ventral horn of the spinal cord, no significant differences were found in the mean (± SD) axon number between the two groups: 344.67 ± 62.3 in Furnas and 322.56 ± 49 in Rabo de Peixe group; *p* = 0.693 (Student’s *t*-test).

**Fig. 4:**
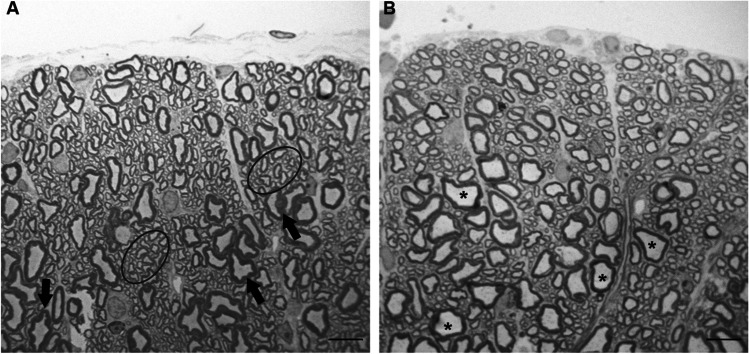
Semithin section of the ventral horn white matter. **A** An animal chronically exposed to volcanic environments. Note the high proportion of small axons (circled area) and the presence of crenated axons (arrows). **B** A mouse from Rabo de Peixe showing larger diameter axons (asterisks); scale bar: 10 μm

At the light microscopic level, the myelinated axons from the two sites were varied in diameter and shape. In addition, some myelinated axons of Furnas mice showed a greater degree of crenation (Fig. 4). It is important to highlight that there was an evident increase of small axons in the individuals from Furnas village compared to the Rabo de Peixe mice, which showed larger diameter axons. Apparently, the largest ones have lost their typical circular shape in both groups (Fig. 4).

Furnas animals showed a significant decrease in axons mean (± SD) diameter (1.55 ± 1.2 μm) compared with those caught in Rabo de Peixe (1.69 ± 1.3 μm), *p* < 0.005 (Wilcoxon Mann–Whitney test).

**Fig. 5:**
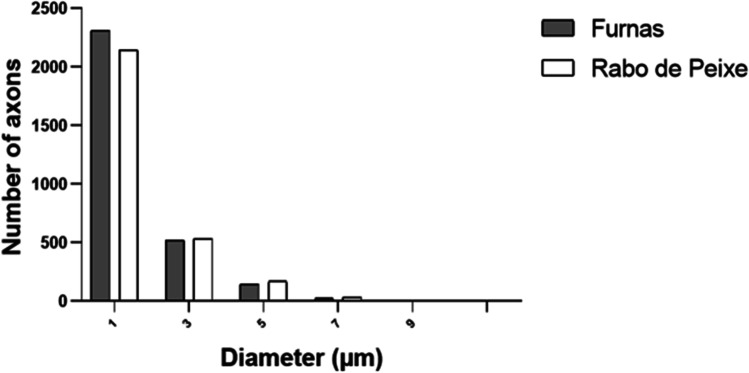
Frequency diameter distribution at a lumbar level of the spinal cord. Observed that there are more small axons in Furnas mice

Also, we tested for the presence of site differences in the distribution of axons with various diameters. Figure 5 provides an overview of the axon-diameter distributions in the two animal groups with non-normally distributed data.

Following Stankovic [33], axons with a calibre ≥ 4 μm were considered large axons. Then, we considered two axon categories depending on diameter: < 4 μm and ≥ 4 μm in diameter axons. Regarding the axon calibre smaller than 4 μm, there were differences in the percentage found between Furnas and Rabo de Peixe rodents. In this sense, in Furnas mice, 94.28% of total axons exhibited diameters smaller than 4 μm, while in Rabo de Peixe, this percentage was 92.28%. Statistical differences were found in the mean (± SD) diameter of both groups: 1.34 ± 0.81 μm in Furnas mice versus 1.42 ± 0.83 μm in Rabo de Peixe animals, *p* < 0.005 (Wilcoxon Mann–Whitney test). Concerning larger axon calibre, both groups presented a small proportion: 5.72% in the exposed rodents and 7.72% in the animals caught in Rabo de Peixe. No significant differences were observed in the axon calibre being greater than 4 μm: 5.09 ± 0.98 μm in the Furnas animals and 5.11 ± 0.98 μm in those of Rabo de Peixe, *p* = 0.619 (Wilcoxon Mann–Whitney test).

Another morphological parameter analyzed was the roundness factor (*R*). Significant differences were observed in the mean (± SD) of the roundness factor between the two studied groups: 1.37 ± 0.36 in the axons from Furnas animals and 1.34 ± 0.35 in the rodents from Rabo de Peixe, *p* < 0.005 (Wilcoxon Mann–Whitney test). Considering that a circular shape implies a roundness factor between 1 and 1.5, a greater proportion of axons with a *R* value similar to a circular shape was observed in Rabo de Peixe animals, 77.67%, than in Furnas animals, 73.38%. These data indicate that exposed individuals to the volcanic environment have axons undergone more atrophic changes.

## Discussion

For the first time, the presence of mercury deposits in the spinal cord of animals inhabiting active volcanic environments has been proven. These findings are consistent with the findings of Navarro-Sempere et al. [26] about the mercury uptake in several locations of the brain.

The link between air pollution and its effects in CNS is widely studied [34–39]. However, little is known about its effects in individuals exposed to active volcanic environments, even though volcanos are relevant sources of contaminants that are harmful to humans [7, 13, 40–42]. Furthermore, the literature focuses on the brain as the main part of the CNS affected by air pollution, often neglecting the spinal cord as an important part of this system.

It is known that active neurons are inclined to take up more circulating toxicants [43], so we have focused our investigation on the lumbar level of the spinal cord since, together with the cervical section, these regions have been described as of greatest motoneuron activity during exercise. Thus, we hypothesized that gaseous mercury (Hg^0^), after inhalation and lung uptake [44], arrives at the skeletal muscle via the bloodstream and is incorporated into the motor neuron through the neuromuscular junction. According to Camarinho et al. [44], the main uptake route Hg^0^ is the lung and, even at very low concentrations in the environment, the chronic exposure to gaseous mercury results in its bioaccumulation in the lung tissue. Once inhaled, Hg^0^ enters the bloodstream, reaching the skeletal muscle and the motor neurons. The Hg^0^ can be oxidized to Hg^++^, mercuric mercury considered the toxic form, by the catalase-hydrogen peroxide pathway [45–47] inside the neurons. Mercury, gaseous or mercuric form, can reach the cell body due to retrograde axonal transport. Our results showed mercury deposits in large neurons of the lumbar spinal cord, which could be motor neurons due to their localization, shape and size. In addition, we found AMG staining in a blood vessel in the ventral fissure suggesting an ongoing exposure to this heavy metal [43]. Therefore, we suppose that inorganic mercury could arrive at motor neurons by the retrograde axonal transport as by the blood vessels that irrigate the spinal cord. However, we did not observe mercury stored in the cell cytoplasm, but this heavy metal adhered to plasma and nuclear membrane as it was noted in the brain of those animals chronically exposed to the active volcanic environment [26] on the island of Sao Miguel. The preference of the Hg for the membranes could be explained due to the amount of sulphhydryl groups to which this heavy metal can bind [48]. Moreover, recent studies show that Hg^0^ can cross the blood–brain barrier [26, 47, 49], revealing that this is probably another important via for the entrance of gaseous mercury in the central nervous system.

The inorganic mercury has been described as an important environmental factor in the pathogenesis of sporadic motor neuron diseases (SMND), including amyotrophic lateral sclerosis (ALS) [50] which is considered the most severe form of the SMND [51]. It has been demonstrated that this form of Hg enters the motor neurons at low doses [52], even if the mercury administered was inhaled [53, 54]. In this respect, our results support this theory since we have found mercury deposits in the motor neurons of those animals exposed to volcanogenic pollutants.

As mentioned above, mercury transport inside the nerve cells could be due to retrograde axonal transport. In this transport phase, heavy metals generate free radicals, which could cause structural damage to neurofilaments through oxidative stress mechanisms. It should be noted that the CNS is very vulnerable to reactive oxygen species and other free radicals. As proposed in the study of Griffin and Watson [55], there is an almost linear relationship between the cross-sectional area and the number of neurofilaments. It can therefore be assumed that if there is damage to the neurofilaments, it will affect the calibre of the axons. In addition, there is evidence that oxidative stress of proteins (such as neurofilaments) occurs both in heavy metal-mediated free radical generation and in the pathophysiology of SMND [56]. Our results evidence this damage since a decline in the axonal calibre was observed in those animals exposed to volcanogenic mercury so that fewer large-calibre axons, as well as a higher number of small axons, were found than in the controls’ mice. However, the number of total axons was similar in both groups. Accordingly, we suggest that there was not a loss of larger neurons but the axoplasm of larger neurons suffered a shrinkage. It should be emphasized that the axonal withdrawal is an important and premature pathological hallmark in the SMND [53]. In addition, larger axons in Furnas animals have shown convoluted forms presenting a greater roundness factor than those mice from the control site. Volcanogenic exposition might affect the turnover of the cytoskeletal matrix responsible for the axonal structure. In this regard, it is known that phosphorylation of both the C-terminal regions and the head domain of neurofilaments contributes to the regulation of the interactions between neurofilaments, neurofilaments with microtubules and the interactions between microtubules and motor proteins, the latter are responsible for axonal transport [57]. These processes may modulate the dynamics of the formation of the neurofilament-based cytoskeletal lattice supporting mature axons [58, 59].

On the other hand, the role of microtubules must be considered since it has been reported that elemental mercury can act as a toxicant also affecting microtubules [60]. It has been shown that mercury can inhibit tubulin polymerization [61], leading to a decrease in microtubule density and disorganization of the axonal cytoskeleton. Due to these factors, the transport of essential neuronal elements could be affected [33]. Therefore, although they do not seem to play an important role in the regulation of axonal diameter, it seems that they are highly involved in fast axonal transport, which is essential for neurons. Further research should be done to investigate the possible ultrastructural changes and the axonal transport in neuron motors of mice inhabiting volcanogenic environments.

In previous studies carried out in the same location in the Azores archipelago, mice living in volcanic environments showed mercury deposits in different locations of the brain [26], as well as reactive astrogliosis in the hippocampal gyrus and astrocytic dysfunction [27]. These findings could indicate that a neuroinflammatory response is occurring and, if sustained over time, it would provoke a scenario of chronic oxidative stress, which could trigger several degenerative diseases [62]. Similarly, the results presented here reflect that this same stress situation could occur in the spinal cord of animals exposed to gaseous mercury, triggering neurodegenerative pathologies, especially those related to the motor system.

Nevertheless, given the complexity of the volcanic environment, several other toxic elements, such as gases and metals, may also contribute to enhancing the development of neural damage since some of these toxic elements may enter the body mainly by inhalation and reach the bloodstream and the CNS. To overcome these limitations, laboratorial essays need to be further developed in order to test the effects of the exposure of genetically controlled animals to each of the main contaminants present in the volcanic environment.

## Conclusions

Overall, these findings support our previous studies about mercury uptake in the CNS. Moreover, we demonstrated changes in axonal calibre and the presence of crenated axons in the mice from Furnas village. Since exposure to mercury and axonal atrophy are risk factors for SMND, we recommend the health surveillance of populations chronically exposed to active volcanic environments.

Finally, it would be important to be able to perform studies in humans since observing any pathological signs of neurodegenerative diseases related to mercury accumulation in mice are very difficult due to their short lifespan.

## Data Availability

The datasets used and/or analyzed during the current study are available from the corresponding author on reasonable request.
